# Genetic architecture and genomic prediction of plant height-related traits in chrysanthemum

**DOI:** 10.1093/hr/uhad236

**Published:** 2023-11-14

**Authors:** Xuefeng Zhang, Jiangshuo Su, Feifei Jia, Yuhua He, Yuan Liao, Zhenxing Wang, Jiafu Jiang, Zhiyong Guan, Weimin Fang, Fadi Chen, Fei Zhang

**Affiliations:** State Key Laboratory of Crop Genetics & Germplasm Enhancement and Utilization, Key Laboratory of Biology of Ornamental Plants in East China, National Forestry and Grassland Administration, College of Horticulture, Nanjing Agricultural University, Nanjing 210095, China; Zhongshan Biological Breeding Laboratory, No. 50 Zhongling Street, Nanjing 210014, China; State Key Laboratory of Crop Genetics & Germplasm Enhancement and Utilization, Key Laboratory of Biology of Ornamental Plants in East China, National Forestry and Grassland Administration, College of Horticulture, Nanjing Agricultural University, Nanjing 210095, China; Zhongshan Biological Breeding Laboratory, No. 50 Zhongling Street, Nanjing 210014, China; State Key Laboratory of Crop Genetics & Germplasm Enhancement and Utilization, Key Laboratory of Biology of Ornamental Plants in East China, National Forestry and Grassland Administration, College of Horticulture, Nanjing Agricultural University, Nanjing 210095, China; State Key Laboratory of Crop Genetics & Germplasm Enhancement and Utilization, Key Laboratory of Biology of Ornamental Plants in East China, National Forestry and Grassland Administration, College of Horticulture, Nanjing Agricultural University, Nanjing 210095, China; Zhongshan Biological Breeding Laboratory, No. 50 Zhongling Street, Nanjing 210014, China; State Key Laboratory of Crop Genetics & Germplasm Enhancement and Utilization, Key Laboratory of Biology of Ornamental Plants in East China, National Forestry and Grassland Administration, College of Horticulture, Nanjing Agricultural University, Nanjing 210095, China; Zhongshan Biological Breeding Laboratory, No. 50 Zhongling Street, Nanjing 210014, China; State Key Laboratory of Crop Genetics & Germplasm Enhancement and Utilization, Key Laboratory of Biology of Ornamental Plants in East China, National Forestry and Grassland Administration, College of Horticulture, Nanjing Agricultural University, Nanjing 210095, China; Zhongshan Biological Breeding Laboratory, No. 50 Zhongling Street, Nanjing 210014, China; State Key Laboratory of Crop Genetics & Germplasm Enhancement and Utilization, Key Laboratory of Biology of Ornamental Plants in East China, National Forestry and Grassland Administration, College of Horticulture, Nanjing Agricultural University, Nanjing 210095, China; Zhongshan Biological Breeding Laboratory, No. 50 Zhongling Street, Nanjing 210014, China; State Key Laboratory of Crop Genetics & Germplasm Enhancement and Utilization, Key Laboratory of Biology of Ornamental Plants in East China, National Forestry and Grassland Administration, College of Horticulture, Nanjing Agricultural University, Nanjing 210095, China; Zhongshan Biological Breeding Laboratory, No. 50 Zhongling Street, Nanjing 210014, China; State Key Laboratory of Crop Genetics & Germplasm Enhancement and Utilization, Key Laboratory of Biology of Ornamental Plants in East China, National Forestry and Grassland Administration, College of Horticulture, Nanjing Agricultural University, Nanjing 210095, China; Zhongshan Biological Breeding Laboratory, No. 50 Zhongling Street, Nanjing 210014, China; State Key Laboratory of Crop Genetics & Germplasm Enhancement and Utilization, Key Laboratory of Biology of Ornamental Plants in East China, National Forestry and Grassland Administration, College of Horticulture, Nanjing Agricultural University, Nanjing 210095, China; Zhongshan Biological Breeding Laboratory, No. 50 Zhongling Street, Nanjing 210014, China; State Key Laboratory of Crop Genetics & Germplasm Enhancement and Utilization, Key Laboratory of Biology of Ornamental Plants in East China, National Forestry and Grassland Administration, College of Horticulture, Nanjing Agricultural University, Nanjing 210095, China; Zhongshan Biological Breeding Laboratory, No. 50 Zhongling Street, Nanjing 210014, China

## Abstract

Plant height (PH) is a crucial trait determining plant architecture in chrysanthemum. To better understand the genetic basis of PH, we investigated the variations of PH, internode number (IN), internode length (IL), and stem diameter (SD) in a panel of 200 cut chrysanthemum accessions. Based on 330 710 high-quality SNPs generated by genotyping by sequencing, a total of 42 associations were identified via a genome-wide association study (GWAS), and 16 genomic regions covering 2.57 Mb of the whole genome were detected through selective sweep analysis. In addition, two SNPs, Chr1_339370594 and Chr18_230810045, respectively associated with PH and SD, overlapped with the selective sweep regions from *F*_ST_ and π ratios. Moreover, candidate genes involved in hormones, growth, transcriptional regulation, and metabolic processes were highlighted based on the annotation of homologous genes in *Arabidopsis* and transcriptomes in chrysanthemum. Finally, genomic selection for four PH-related traits was performed using a ridge regression best linear unbiased predictor model (rrBLUP) and six marker sets. The marker set constituting the top 1000 most significant SNPs identified via GWAS showed higher predictabilities for the four PH-related traits, ranging from 0.94 to 0.97. These findings improve our knowledge of the genetic basis of PH and provide valuable markers that could be applied in chrysanthemum genomic selection breeding programs.

## Introduction

Plant height (PH), limited by stem elongation, is one of the essential traits determining plant architecture and plays a crucial role in light interception and photosynthesis in the canopy [1]. Moreover, PH is the primary factor affecting crops’ lodging resistance and seed/fruit yields, targeted in crop domestication. The best proof is that the green revolution introduced the semi-dwarf phenotype into crops to improve yield [2]. However, during modern breeding, the requirements of breeders for PH are not immutable and frozen, but mainly depend on diverse environments and cultivation practices. Hence, understanding the genetic basis of PH will contribute to cultivating the ideal plant types to improve agronomic traits.

Chrysanthemum is an economically important ornamental plant because of its rich genetic diversity in flower type, flower color, and plant architecture, and it ranks second in the world flower industry. PH is relevant to cut chrysanthemum quality. The stem of cut chrysanthemums should reach a harvesting standard of 80 cm in length. Too high PH increases production energy consumption and the likelihood of lodging in the cultivation process; nevertheless, too short PH adversely affects flower quality. Therefore, the ideal PH has been regarded as an essential objective in modern chrysanthemum breeding. In addition, internode number (IN), internode length (IL) and stem diameter (SD), as the main factors of PH, have significant phenotypic correlation with PH, and are also important indicators of the ideal plant types of cut chrysanthemum. Previous studies confirmed the quantitative inheritance of PH, with moderately high heritability in chrysanthemum. Zhang *et al*. [3] dissected the additive and epistatic effects by genetic mapping in a chrysanthemum segregating *F*_1_ population. Klie *et al*. [4] reported several significant associations for PH in cut chrysanthemums. However, previous linkage mapping and association analysis depended mainly on traditional PCR-based markers in chrysanthemums. It is an arduous challenge to localize candidate genes for PH due to the limited number of markers and lack of genome information.

In the last decades, quantitative trait loci (QTLs) and candidate genes underlying PH were identified in different plants by continuously updating and improving molecular markers, locating populations, and statistical methods. Of these, genome-wide association study (GWAS) is a powerful approach to elucidating the genetic basis and identifying alleles/genes related to complex quantitative traits at the population level. Assefa *et al*. [5] identified additive and epistatic loci possibly governing PH via GWAS and genome-wide epistatic study in soybean, and identified a candidate gene, *Dt1*, responsible for regulating PH and flower development. Lu *et al*. [6] detected significantly associated SNPs for PH in maize inbred lines via GWAS, and mined a candidate gene, *GRMZM2G164265*, that encodes a kinase-related protein and functions in PH regulation. Ma *et al*. [1] identified 10 QTLs and a novel gene, *GhPIN3*, for PH by linkage mapping in a backcross inbred line (BIL) population of cotton. Currently, the relevant QTLs and genetic pathways for PH have been well elucidated in most major crops, shedding light on the genetic architecture of PH for improving breeding efficiency. However, PH’s inheritance determinism remains largely unknown in ornamental crops. Accompanying GWAS, selective sweep analysis is an alternative for evaluating the degree of genetic differentiation between genetic subpopulations and screening putative genomic regions under selection pressure caused by domestication or artificial selection. Chong *et al*. [7] deduced that the cultivated chrysanthemum experienced strong and extensive artificial selection in the process of domestication and improvement by comparing the differentiated genomic regions of diverse types of chrysanthemums. Contrary to GWAS and selective sweep analysis, genomic selection (GS) does not need to determine significant associations in advance but directly uses genome-wide markers to evaluate genomic estimated breeding values (GEBVs). It is reported that GS is promising in selecting complex traits controlled by numerous small-effect genes at plants’ early growth stage and shortening the breeding cycle [8].

The reference genome of cultivated chrysanthemums has lately been released [9], laying an indispensable foundation for elucidating the genetic determinism of chrysanthemums. Hence, it is essential to integrate genomic tools that can promote accurate selection and insight into the molecular basis of essential traits such as PH. The objectives of the current study were to uncover the genetic basis and identify the marker–trait associations and candidate genes for chrysanthemum PH via combined GWAS and selective sweep analysis and to explore the possibility of GS for selecting PH in breeding programs. Results from this study provide new insights into the genetic architecture of chrysanthemum PH for improving future selection breeding.

## Results

### Phenotypic analysis

**Figure 1:**
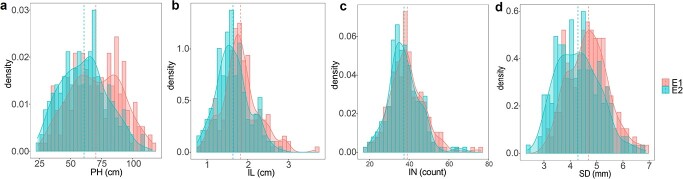
Distribution of PH (**a**), IL (**b**), IN (**c**), and SD (**d**) in two environments (E1 and E2). The dashed lines represent the mean values of the traits.

Extensive variations for PH-related traits were observed in the 200 chrysanthemum accessions under two environments (Fig. 1, Supplementary Data Table S3). High correlations were calculated for PH-related traits between the two environments, the correlation coefficient ranging from 0.43 to 0.58 (Supplementary Data Fig. S1). PH had the highest coefficient of variation of 30.18% in E2, ranging from 23.58 to 109.20 cm, with an average of 61.69 cm (Table 1). Compared with other traits, SD had the lowest coefficient of variation in E1 and E2, with a coefficient of variation of 16.49 and 18.88%, respectively (Table 1). There were significant correlations among the four PH-related traits (Supplementary Data Table S4). PH is mainly determined by IN and IL, so it is unsurprising that PH positively correlates with IN and IL. Analysis of variance showed that genotype, environment, and genotype × environment interaction significantly affected the four PH-related traits (Supplementary Data Table S5). The *H*^2^ of PH, IL, IN, and SD was 79.61, 84.42, 86.06, and 79.21%, respectively (Table 1). The results suggested that, despite environmental effects, genetic effects play a crucial role in the expression of chrysanthemum PH-related traits.

**Table 1:** Phenotypic variations for chrysanthemum PH-related traits.

**Traits**	**Environment**	**Mean**	**Maximum**	**Minimum**	**SD** ^**a**^	**CV** ^**b**^ **(%)**	***H*** ^**2**^ ^**c**^ **(%)**
PH (cm)	E1	70.65	119.28	27.53	20.67	29.26	79.61
	E2	61.69	109.20	23.58	18.62	30.18	
IL (cm)	E1	1.84	3.80	0.78	0.49	26.63	84.42
	E2	1.66	3.35	0.76	0.42	25.30	
IN (count)	E1	38.87	76.20	18.80	8.90	22.90	86.06
	E2	37.36	67.80	19.00	7.92	21.20	
SD (mm)	E1	4.67	6.87	2.77	0.77	16.49	79.21
	E2	4.29	6.82	2.37	0.81	18.88	

a Standard deviation;

b coefficient of variation;

c broad-sense heritability.

### Genetic structure analysis

Based on 330 710 high-quality SNPs, the PCA demonstrated that the 200 chrysanthemum accessions did not exhibit significant genetic differentiation, which meets the requirements of association analysis (Supplementary Data Fig. S2a). The kinship heat map also showed overall relative weak relationships among different chrysanthemum genotypes (Supplementary Data Fig. S2b), with average coefficient of kinship of 0.02, indicating the existence of extensive genetic diversity in the population.

### Genome-wide association analysis

**Figure 2:**
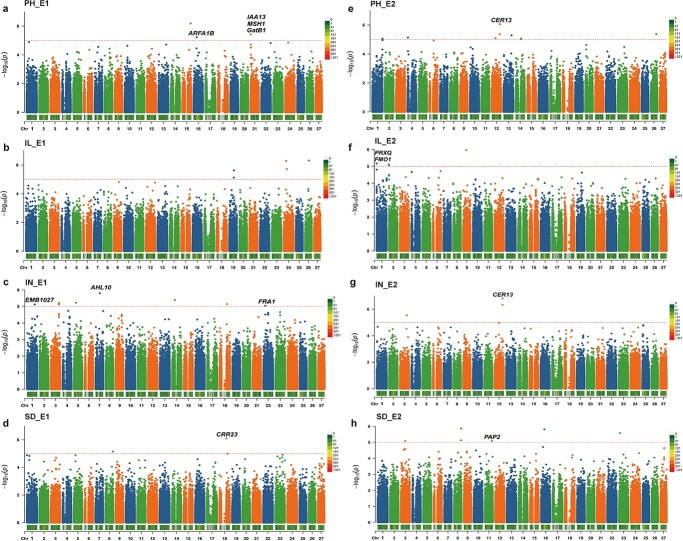
Manhattan plots of GWAS for PH-related traits in E1 (**a**–**d**) and E2 (**e**–**h**). The dashed line represents the significance threshold (−log10(*P*) = 5), and proposed candidate genes for PH-related traits are marked above the corresponding SNPs.

GWAS was performed using the PCA + K model in EMMAX. As a result, a total of 42 significant SNPs were associated with PH-related traits in two environments under the threshold of *P* < 1 × 10^−5^, of which 18 SNPs passed genome-wide Bonferroni correction (*P* < 4.16E−06; Fig. 2, Supplementary Data Fig. S3). For PH, 12 significant SNPs were detected, located on chromosomes 1, 4, 12, 13, 14, 15, 16, 21, and 26, respectively (Table 2, Fig. 2). Of these, three SNPs (Chr15_281792329, Chr16_103728540, and Chr21_9532188) were detected in E1, while the remaining nine SNPs were detected in E2. The significant SNPs showed positive phenotypic effects except for the two SNPs Chr16_103728540 and Chr21_9532188 (Table 2). For IL, we detected 10 significant SNPs, including five each in E1 and E2, with phenotypic effects ranging from 0.2 to 0.45 cm (Table 2). For IN, 12 significant SNPs were identified, of which only two (Chr3_233732224 and Chr12_270808434) were from E2, with phenotypic effects ranging from −9.27 to 8.95 (Table 2). Additionally, we identified two and six significant SNPs for SD in E1 and E2, respectively, located on chromosomes 3, 8, 11, 16, 18, and 23, with only one SNP, Chr8_245595683, showing a negative phenotypic effect (Table 2). Regretfully, no common SNP was detected across the two environments. However, we observed that the SNPs Chr12_270808434 and Chr12_270808478 were simultaneously detected for PH and IN, both showing positive effects on PH and IN. In particular, Chr12_270808434 remained significant after employing the Bonferroni correction.

**Table 2:** Significant SNPs associated with PH-related traits.

**Traits**	**SNP** ^**a**^	**Position (bp)**	**−Log**_**10**_ **(*P*)**	**Allele** ^**b**^	**MAF** ^**c**^	***a***_***i***_ ^**d**^	**Environment**
PH	Chr1_339370594	339 370 594	5.06	G/T	0.20	10.60^**^	E2
	Chr4_13976903	13 976 903	5.13	A/G	0.23	11.25^**^	E2
	Chr12_124163766	124 163 766	5.09	A/T	0.22	8.20^**^	E2
	**Chr12_270808434**	270 808 434	6.06	A/G	0.40	11.97^**^	E2
	Chr12_270808478	270 808 478	5.36	G/A	0.40	10.83^**^	E2
	Chr13_269150736	269 150 736	5.29	G/A	0.15	11.27^**^	E2
	Chr14_183595760	183 595 760	5.03	G/A	0.10	11.92^**^	E2
	Chr14_183595863	183 595 863	5.06	A/T	0.10	12.10^**^	E2
	**Chr15_281792329**	281 792 329	6.18	T/A	0.06	12.12^*^	E1
	Chr16_103728540	103 728 540	5.22	T/G	0.06	−13.06^**^	E1
	**Chr21_9532188**	9 532 188	5.42	G/T	0.10	−10.43^**^	E1
	**Chr26_184971991**	184 971 991	5.38	C/A	0.10	17.40^**^	E2
IL	Chr1_1200392	1 200 392	5.17	C/T	0.16	0.20^*^	E2
	Chr2_345676	345 676	5.12	T/A	0.22	0.20^**^	E2
	Chr2_345792	345 792	5.07	A/G	0.22	0.20^**^	E2
	Chr2_345978	345 978	5.08	C/T	0.20	0.23^**^	E2
	**Chr9_110756763**	110 756 763	5.96	C/A	0.12	0.28^**^	E2
	**Chr19_155208240**	155 208 240	5.63	C/A	0.08	0.31^**^	E1
	Chr19_155208347	155 208 347	5.12	G/A	0.09	0.29^**^	E1
	**Chr24_105773942**	105 773 942	5.71	G/T	0.09	0.45^**^	E1
	**Chr24_82362276**	82 362 276	6.28	C/A	0.07	0.40^**^	E1
	**Chr26_124735601**	124 735 601	6.32	G/A	0.06	0.29^**^	E1
IN	Chr1_255259266	255 259 266	5.12	C/T	0.21	−5.91^**^	E1
	Chr3_264364249	264 364 249	5.12	G/A	0.36	2.93^ns^	E1
	Chr3_264364290	264 364 290	5.21	C/T	0.36	2.89^ns^	E1
	**Chr3_233732224**	233 732 224	5.55	A/T	0.23	−4.69^**^	E2
	Chr5_70145757	70 145 757	5.22	G/A	0.06	8.95^*^	E1
	**Chr7_142340238**	142 340 238	5.80	G/A	0.26	−5.78^**^	E1
	**Chr12_270808434**	270 808 434	6.80	A/G	0.40	6.34^**^	E2
	**Chr12_270808478**	270 808 478	6.33	G/A	0.40	5.11^**^	E1
	**Chr14_88122819**	88 122 819	5.39	G/T	0.12	6.80^**^	E1
	Chr18_244242678	244 242 678	5.14	C/T	0.16	3.58^*^	E1
	Chr22_61447919	61 447 919	5.23	C/T	0.43	−9.27^**^	E1
	Chr22_153548117	153 548 117	5.04	C/T	0.18	6.77^**^	E1
SD	Chr3_149866685	149 866 685	5.09	T/G	0.12	0.59^**^	E2
	Chr8_245595641	245 595 641	5.15	G/T	0.29	0.47^**^	E2
	**Chr8_245595683**	245 595 683	5.86	A/G	0.42	−0.52^**^	E2
	Chr8_246318480	246 318 480	5.13	C/T	0.20	0.45^**^	E1
	Chr11_239625612	239 625 612	5.09	A/G	0.49	0.78^**^	E2
	**Chr16_113771349**	113 771 349	5.81	T/G	0.06	0.96^**^	E2
	**Chr18_230810045**	230 810 045	6.11	A/T	0.20	0.68^**^	E1
	**Chr23_35995783**	35 995 783	5.58	G/T	0.07	0.81^**^	E2

a Bold type indicates that *P* values were less than the Bonferroni correction (*P* = 4.16E−06);

b major alleles/minor alleles;

c minor allele frequency;

d *a_i_* represents phenotypic effects; ^*^P < 0.05; ^**^P < 0.01; ns, not significant.

### Prediction of candidate genes

To identify candidate genes for PH-related traits, we mined 118 genes within an interval of 200 kb for the GWAS-derived 42 significant SNPs by combining gene function annotation in *Arabidopsis* and chrysanthemum transcriptome data. Nearly three-quarters (29/42) of the SNPs had at least two genes (Supplementary Data Table S6). Because PH-related traits are mainly determined by the growth and development of the stem, we focused on the genes that showed a tendency for higher expression in shoot apexes (Supplementary Data Fig. S4). As a result, 19 genes associated with PH-related traits were selected as potential candidate genes (Table 3, Supplementary Data Fig. S4).

**Table 3:** Information on proposed candidate genes for PH-related traits identified by GWAS.

**Traits**	**SNP**	**Candidate gene**	**Chromosome**	**Gene start (bp)**	**Gene end (bp)**	***Arabidopsis* ortholog**	**Gene annotation**
PH	Chr12_270808434	*evm.model.scaffold_1205.255*	Chr12	270 770 208	270 774 229	*CER13*	Involved in alkane biosynthetic process and seed development
	Chr16_103728540	*evm.model.scaffold_87.144*	Chr16	103 675 400	103 679 963	*ARFA1B*	Vesicle-mediated transport
	Chr21_9532188	*evm.model.scaffold_985.228*	Chr21	9 519 219	9 521 647	*IAA13*	Response to auxin
	Chr21_9532188	*evm.model.scaffold_985.232.4*	Chr21	9 540 972	9 549 797	*MSH1*	Encodes a DNA-binding protein
	Chr21_9532188	*evm.model.scaffold_985.234*	Chr21	9 557 172	9 562 052	*GatB*	Encodes Glu-tRNA (Gln) amido transferase subunit B
	Chr26_184971991	*evm.model.scaffold_1656.18*	Chr26	185 014 610	185 016 676	NA	Associated with shoot system development
	Chr26_184971991	*evm.model.scaffold_1656.9*	Chr26	184 889 376	184 894 098	NA	MATE efflux family protein
IL	Chr1_1200392	*evm.model.scaffold_6916.84*	Chr1	1 257 799	1 259 139	*PRXQ*	Involved in cell redox homeostasis
	Chr1_1200392	*evm.model.scaffold_6916.85*	Chr1	1 259 141	1 261 491	*FMO1*	Involved in excess light stress-induced signal transduction and defense response to bacteria and fungi
	Chr19_155208240	*evm.model.scaffold_1046.546*	Chr19	155 205 355	155 208 408	NA	Involved in positive regulation of ubiquitin-dependent protein catabolic process
	Chr24_82362276	*evm.model.scaffold_764.63*	Chr24	82 445 864	82 448 604	NA	Involved in methylation
IN	Chr1_255259266	*evm.model.scaffold_1254.22*	Chr1	255 223 557	255 232 321	*EMB1027*	Involved in embryo development ending
	Chr7_142340238	*evm.model.scaffold_841.11*	Chr7	142 397 744	142 401 139	*AHL10*	Involved in growth with stress and defense responses
	Chr18_244242678	*evm.model.scaffold_1597.67*	Chr18	244 196 540	244 198 436	NA	Ribosomal protein
	Chr22_153548117	*evm.model.scaffold_1826.23*	Chr22	153 513 398	153 515 294	NA	Transmembrane protein
	Chr22_61447919	*evm.model.scaffold_247.307*	Chr22	61 444 432	61 452 371	*FRA1*	Involved in plant-type cell wall biogenesis
SD	Chr11_239625612	*evm.model.scaffold_1760.109*	Chr11	239 603 354	239 607 017	*PAP2*	Purple acid phosphatases superfamily protein
	Chr18_230810045	*evm.model.scaffold_197.213*	Chr18	230 877 970	230 879 425	*CRR23*	Involved in PSI cyclic electron transport
	Chr18_230810045	*evm.model.scaffold_197.216*	Chr18	230 908 743	230 915 171	NA	ARM repeat superfamily protein

NA indicates that the *Arabidopsis* homolog of this candidate gene has not been found.

**Figure 3:**
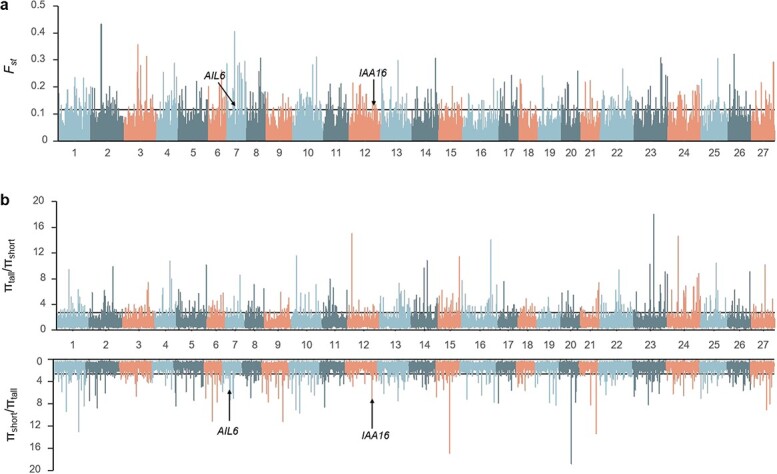
Genome-wide selective sweep analysis by comparing the tall and short groups. The horizontal lines represent the threshold values of the top 1% of *F*_ST_ (**a**) and π ratios (**b**). Candidate genes related to hormones and highly expressed in shoot apexes are marked with arrows.

**Figure 4:**
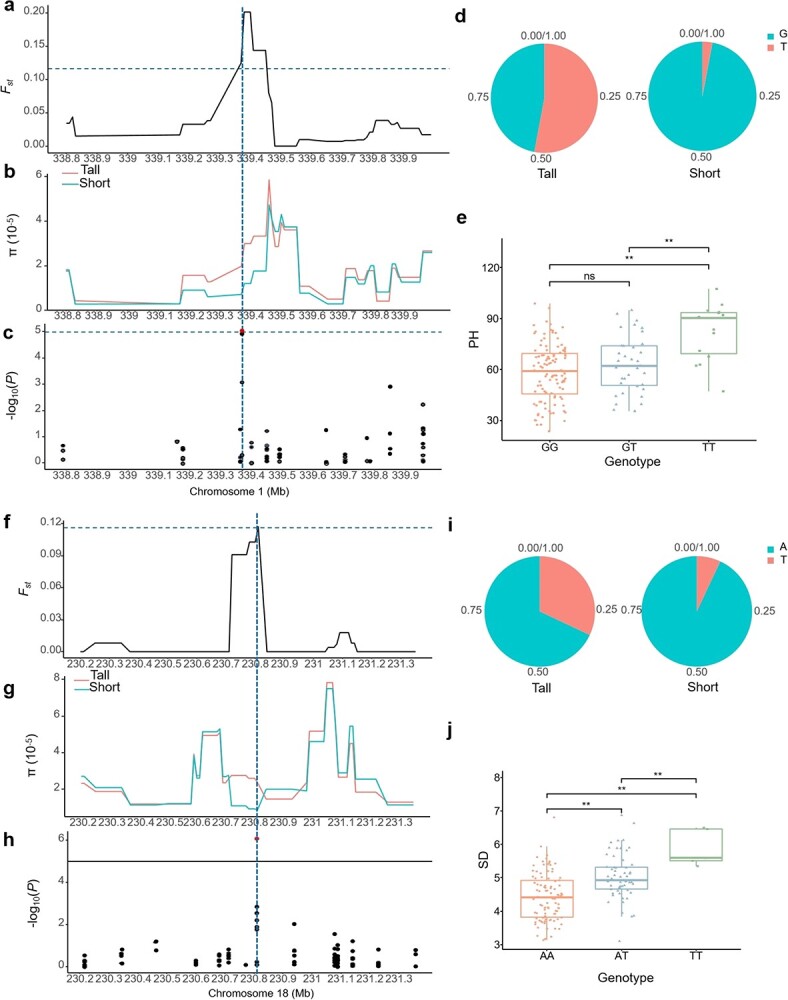
Selective signature and phenotypic difference between individuals with different alleles of Chr1_33937059 associated with PH (**a**–**e**) and Chr18_230810045 associated with SD (**f**–**j**). **a**, **f***F*_ST_ values of the tall group versus the short group. **b**, **g** Distribution of π in the tall and short group of the 1.1 Mb genetic regions surrounding the SNP. **c**, **h** Significant GWAS signals coincident with the selective sweep regions. **d**, **i** Allele frequencies of the SNP between the tall and short groups. **e**, **j** Boxplot of PH and SD in accessions with different genotypes. ^*^P < 0.05; ^**^P < 0.01; ns not significant.

**Figure 5:**
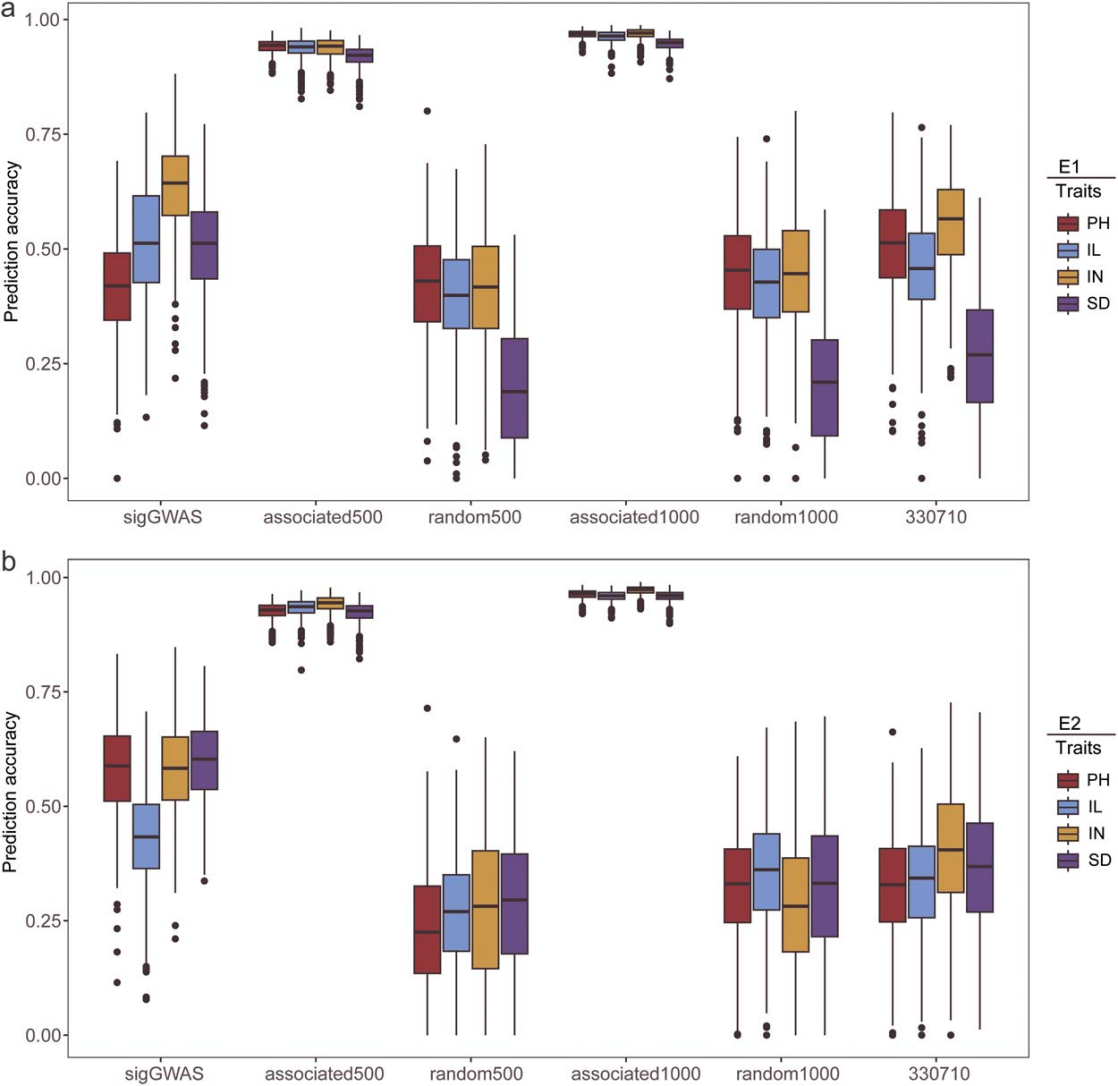
Genomic prediction accuracy for the four PH-related traits evaluated using the rrBLUP model in E1 (**a**) and E2 (**b**).

For PH, we noted several candidate genes having functional annotations related to cell polarity, cell division, auxin, and plant growth (Table 3). *evm.model.scaffold_1205.255*, involved in alkane biosynthetic process and seed development, encodes an auxin response factor (*OsARF18*) in rice [27]. On chromosome 16, a candidate gene, *evm.model.scaffold_87.144*, is homologous to *ARFA1B*, which functions in epidermal cell polarity [28]. On chromosome 21, three candidate genes were identified in the regions around Chr21_9532188. One candidate gene, *evm.model.scaffold_985.228*, is homologous to auxin-induced gene *IAA13*, involved in responding to auxin, and plays an important role in lateral root initiation in rice [29, 30]. One candidate gene, *evm.model.scaffold_985.232.4*, 19.33 kb away from *evm.model.scaffold_985.228*, is homologous to *MSH1*, encoding a DNA-binding protein that may produce heritable changes in plant growth in *Arabidopsis* and tomato [31]. Another candidate gene, *evm.model.scaffold_985.234*, 35.53 kb away from *evm.model.scaffold_985.228*, is homologous to *GatB* involved in Gln-tRNA^Gln^ formation in mitochondria, playing essential roles in determining cell division in rice [32]. On chromosome 26, candidate gene *evm.model.scaffold_1656.18* is annotated as associated with shoot system development and regulation of the developmental process. In addition, a potential association network containing association loci and candidate genes for PH has been demonstrated (Supplementary Data Fig. S5).

For IL, the candidate gene *evm.model.scaffold_6916.84* is homologous to *PRXQ*. It encodes peroxiredoxin Q decomposing peroxides and plays a role in protecting the photosynthetic apparatus to stabilize early plant growth and development [33].

For IN, we mainly noted the candidate genes having functional annotations related to auxin, brassinosteroid, and cell wall biogenesis (Table 3). On chromosome 7, the candidate gene *evm.model.scaffold_841.11* was identified downstream of Chr7_142340238, involved in stress growth regulation and plant organ morphogenesis [34, 35]. On chromosome 22, the candidate gene *evm.model.scaffold_247.307*, containing the SNP Chr22_61447919 located in the exon, encodes a kinesin-like protein with an N-terminal microtubule binding motor domain and mediates cell elongation in rice [36] and cotton [37].

For SD, on chromosome 11, one candidate gene, *evm.model.scaffold_1760.109*, is homologous to *PAP2*, which encodes a dual-localized acid phosphatase (mitochondria and chloroplasts) that modulates carbon metabolism and affects plant growth in tomato [38] and *Camelina sativa* [39].

To prove the relationship between PH and candidate genes, qRT–PCR was carried out on the candidate genes associated with PH that we focused on. The relative expression levels of three candidate genes—*ARFA1B*, *MSH1*, and *IAA13*—in the three tall accessions were significantly higher than that in the three short accessions (*P <* 0.01; Supplementary Data Fig. S6), suggesting these genes might be positively related to PH. Furthermore, we genotyped the SNPs (Chr16_103728540 and Chr21_9532188) corresponding to these three candidate genes identified by qRT–PCR. For the SNP Chr16_103728540, the PH of accessions with genotype GT (56.60 cm) was significantly lower than that of accessions with genotype TT (66.95 cm) (*P <* 0.01; Supplementary Data Fig. S7). For the SNP Chr21_9532188, the PH of accessions with genotype GT (59.65 cm) was significantly lower than that of accessions with genotype GG (67.14 cm) (*P <* 0.05; Supplementary Data Fig. S7).

### Differentially selective signals

Because favorable alleles may be targeted in the breeding process, we tried to perform selective sweep analysis to identify the potential selective signals for PH-related traits. The tall and short groups were used to identify indicators of selective signals, including *F*_ST_ and π ratios (Fig. 3). A total of 130 differentiated genomic regions covering 19.09 Mb of the whole genome were detected, with a top 1% selective signal cut-off (Supplementary Data Table S7). Interestingly, we discovered that a selective sweep signal with the top 1% of *F*_ST_ values on chromosome 1 overlapped with the significant SNP (Chr1_339370594) associated with PH (Fig. 4a and c). Furthermore, the π value of the selective sweep signal in the short group was much lower than that in the tall group (Fig. 4b). We then compared the allele frequency of the significant SNP between the tall and short groups and found that the allele T in the tall group accounted for 53%, much higher than 3% in the short group (Fig. 4d). Meanwhile, based on the whole panel, we observed that the PH of accessions with genotype TT was 83.13 cm, which was significantly higher than 62.58 cm in accessions with genotype GG (Fig. 4e). In addition, another selective sweep signal with the top 1% of *F*_ST_ values and π_tall_/π_short_ values overlapped with an association (Chr18_230810045) identified for SD, which had a lower π value in the short group than in the tall group (Fig. 4f–h). The frequency of allele T in the tall group was 32%, greater than 7% in the short group (Fig. 4i). In the whole panel, the SD of accessions with genotype TT (5.8 mm) was significantly greater than that of accessions with genotype AA (4.43 mm) (Fig. 4j). These results illustrate that the SNPs Chr1_339370594 and Chr18_230810045 are associated considerably with PH-related traits, which might be selected in the process of chrysanthemum breeding. To further exclude false-positive rates, we then recruited XP-CLR analysis to define potential selective sweeps. Sixteen of the 130 differentiated genomic regions were shared by three approaches (Supplementary Data Table S8), covering 2.57 Mb of the whole genome and containing 221 genes. These genes were mainly enriched in biological processes through GO enrichment analysis, including cellular, metabolic, signaling, and developmental processes (Supplementary Data Table S9, Supplementary Data Fig. S8). We speculate that these genes may be involved in the domestication of chrysanthemum for PH.

### Genomic prediction of PH-related traits

Genomic prediction of PH-related traits was carried out with 5-fold cross-validation using the rrBLUP model and different marker sets (Fig. 5, Supplementary Data Table S10). The prediction accuracy of the 330 710 SNPs for PH-related traits was significantly lower than that of sigGWAS, associated500, and associated1000 SNPs under the two environments, except for PH in E1. The prediction accuracy of the random1000 SNPs for PH-related traits was higher than that of the random500 SNPs in the two environments. In addition, the prediction accuracy of the two random SNP sets was significantly lower than that of the two corresponding associated SNP sets, and the prediction accuracy of the sigGWAS SNPs containing 8–12 marker–trait associations was higher than or close to that of the random1000 and random500 SNPs, highlighting the efficiency of significant SNPs in the prediction of PH-related traits. Whether in E1 or E2, the associated1000 SNPs exhibited the highest prediction accuracy for four PH-related traits, with a range of 0.94–0.97. Thus the associated1000 marker set was more suitable for genomic selection. Meanwhile, we observed that the prediction accuracy of IN was slightly higher than that of other traits with different marker sets in the two environments.

## Discussion

PH is one of the crucial components of plant architecture, affecting chrysanthemum quality and production efficiency. In chrysanthemum cultivation, plant growth regulators are often used to control the PH, which consumes a lot of human and material resources and increases potential risks to the environment and human health. Breeders need to pay more attention to plant architecture in chrysanthemums; however, the genetics of PH have only been reported sporadically. In this study, the PH-related traits were highly variable among the investigated chrysanthemum accessions. According to the considerable variances for genotype, environment, and genotype × environment interaction, we conclude that both additive and epistatic effects determined the expression of PH-related traits. The broad-sense heritabilities were calculated at 79–86%, higher than ~69% recorded for PH in a segregating *F*_1_ population of chrysanthemum [3], indicating a sizeable genetic potential. Significant correlations between the two field experiments, along with the relatively high heritability estimates, demonstrate that the field experiments are repeatable and reproducible.

In chrysanthemums, GWAS has been introduced to understand the genetic architecture and identify favorable alleles for traits of interest, including inflorescence [40], aphid resistance [41], drought tolerance [42], and waterlogging tolerance [43]. However, due to the small population size, the limited number of traditional PCR-based markers, and the need for complete genome information, it is quite challenging to successfully implement candidate gene mining or marker-assisted selection breeding in these studies. With the development of DNA sequencing, next-generation sequencing provides large-scale, high-throughput, and low-cost SNPs, paving the way to genetically improving important traits. Lately, the chromosome-scale genome assembly of cultivated chrysanthemum has facilitated genetic research regarding complex traits of ornamental and economic importance [9]. Based on 330 710 GBS-based SNPs in our present study, GWAS generated 42 SNPs significantly associated with PH-related traits, including 20 SNPs from E1 and 22 from E2. However, no common SNP was simultaneously detected across the two environments. Even if the information about phenotype and genotype is entirely accurate, some associations may not be repeated due to the interaction between genotype and environment [44]. It can be seen that the interaction between genotype and environment plays an essential role in individual genetic variation, and so do traits with high heritability. Therefore, the SNPs still need to be verified by evaluating PH-related traits in more populations and environments. Notably, the SNPs Chr12_270808434 and Chr12_270808478 were detected simultaneously for PH and IN and showed a positive effect. Previous studies also showed a significant phenotypic correlation between PH and IN and one locus associated with both PH and IN in soybean [5, 45]. Therefore, we hypothesize that these traits may be controlled by a pleiotropic gene or closely linked loci.

Selective sweep analysis is a crucial tool to identify genes with significant differences in allelic frequencies, which may be used as a complement to GWAS. Sun *et al*. [46] detected multiple GWAS loci located in selective sweep signals between two groups with different PH in rapeseed, providing evidence supporting the GWAS results. Zhang *et al*. [47] identified multiple selective intervals implicated by linkage studies that affected PH in sorghum, but the intervals harbor few GWAS loci. Here, to determine the genomic regions responsive to selection for PH, by comparing the selection signal indicators, *F*_ST_ , π ratios, and XP-CLR we identified a total of 16 overlapping selective regions covering 2.57 Mb of the whole genome. Interestingly, by overlapping the results of *F*_ST_ and π ratios, we found that one selective region overlapped with the GWAS signature (Chr18_230810045) for SD and one selective region harbored a GWAS signal (Chr1_339370594) associated with PH. However, most GWAS signals do not overlap with selective regions, and the areas carrying GWAS signals had *F*_ST_ and π ratios less than the predefined threshold values. On the one hand, we suppose that breeders may have paid more attention to flower type, stress resistance, and quality attributes than PH in previous breeding activities, so there were few strong selection signals associated with PH in the genome. On the other hand, it may be due to the existence of potential confounding effects of population structure in the two groups used for selective sweep analysis, which cannot clearly reflect the selection history of PH. Therefore, we need to employ traditional breeding methods to create more representative populations with fewer confounding effects and significant differences in PH for further verification. It is worth pointing out that due to GBS’s limitation of low coverage, whole-genome-wide SNP markers using resequencing will be required to reveal more selective regions for PH in the future.

As a quantitative trait controlled by multiple genes, PH has relatively complex genetic regulatory networks, which dramatically hinders the mining of candidate genes. In the model plants *Arabidopsis* and rice, most genes regulating PH are related to the anabolism and signal transduction of hormones, including gibberellin (GA), brassinosteroid, and auxin [48]. Research has shown that the genetic path regulating the height of flowering plants is relatively conservative [49], which provides a basis for us to mine candidate genes related to PH in chrysanthemum. Petty *et al*. [50] reported that the expression of the *A. thaliana gai* (*gibberellic acid insensitive*) gene under its promoter could reduce chrysanthemum PH by weakening the response of gibberellin. Suh *et al*. [51] overexpressed *Brassica rapa SRS7* (*short internode related sequence 7*) in pot-type chrysanthemum, which was known to regulate stem size in Chinese cabbage, and discovered that *SRS7* expression was associated with expression of GA- and auxin-related genes. Recently, Zhang *et al*. [52] clarified that the non-polar YAB transcription factor *CmDRP* regulates chrysanthemum PH by directly inhibiting the expression of the GA biosynthesis gene *CmGA3ox*, thereby affecting the biosynthesis of GA. This study identified 32 candidate genes related to PH through GWAS, including an auxin-induced gene *IAA13* (Supplementary Data Table S4). *IAA13* encodes a short-lived nuclear-localized protein belonging to the Aux/IAA gene family, whose relative expression was significantly higher in tall accessions than in short accessions (Supplementary Data Fig. S4). Subsequently, several genes functionally associated with hormones were identified within the potential selective sweeps. *IAA16* is also a gene responding to auxin, and the *iaa16-1* mutation reduced the auxin response, significantly inhibited the growth of plants, and eliminated fertility when homozygous [53]. *AIL6* encodes an AP2-domain transcription factor, regulating shoot apical meristem function and modulating local auxin production in the shoot apical meristem [54]. We also identified several novel candidate genes involved in various pathways, including vesicle-mediated transport, transcriptional regulation, metabolism, methylation, redox, and growth with stress. Although no reports demonstrate that these genes are directly related to the regulation of PH development, the candidate genes underlying associated loci through forward genetics provide important genetic resources for further research on chrysanthemum PH breeding.

Chrysanthemum is a complex species with high heterozygosity (>3%), high repetitiveness (>80%), huge genome size (8.47–9.02 Gb) [9], rapid decay rate, and a relatively low gene density of approximately one gene per 58.74 kb, which makes it rather challenging to identify causal genes underlying the detected association loci within a relatively narrow genomic region. It has been documented that a wide range of 100 kb region centered on the peak SNPs has been employed to explore candidate genes in species with rapid decay rate, such as ginkgo [55] and apple [56]. Therefore, to overcome the challenge of candidate gene identification in chrysanthemum GWAS, a relatively less stringent range of 100 kb upstream and downstream of the significant SNPs was first used to detect the putative candidate genes, and then we combined the RNA-seq data and qRT–PCR analyses to verify the candidate genes responsible for PH traits. In the current work, three candidate genes with differential expression, *ARFA1B*, *MSH1*, and *IAA13*, located at distances of 48, 10, and 8 kb from the associations, respectively, were highlighted, to some extent confirming the reliability of our GWAS results. With the decreasing cost of sequencing and the recent availability of chrysanthemum genome information, denser markers and larger sample sizes are imperative for accurate physical interval demarcation in future chrysanthemum GWAS.

In the past decade, GS has been introduced into several horticultural and agronomic plants [57–59]. Successful implementation of GS depends on prediction accuracy, which is influenced by genetic and statistical factors. The genetic factors include heritability, sample size, marker density, etc. In our study, the rrBLUP model was used for GS of PH-related traits, which performs excellently in predicting most agronomic traits controlled by a large number of QTLs with small effects. Prediction accuracy for IN was slightly higher than that for PH, IL, and SD, possibly due to its higher heritability. Similarly, the prediction accuracy for drought tolerance indices (low heritability) was higher than that for flowering time (high heritability) in the MAGIC cowpea population [60]. This is consistent with the conclusion that, under ideal conditions, traits with high heritability are positively correlated with higher GEBV prediction [61]. Additionally, GS was conducted using six different marker sets to identify a subset of fewer markers that can effectively predict breeding value. Compared with the randomly selected SNP sets, the marker sets containing SNPs associated with PH-related traits displayed an improved prediction accuracy of GS, among which the associated1000 marker set showed the highest accuracies, 0.94–0.97. Therefore, the application of GS can help better select PH-related traits at earlier stages in practical breeding programs.

Taken together, the findings of the current study add deep insights into the genetic architecture of chrysanthemum PH-related traits, and the detected associations, candidate genes, and GS enable efficient selection for genotypes with a desired PH. Furthermore, the domestication of PH can be further investigated by the use of more diversified populations, and the biological function of candidate genes identified herein should be explored in future research.

## Materials and methods

### Plant materials and experimental design

A total of 200 chrysanthemum accessions with apparent differences in PH were obtained from the Chrysanthemum Germplasm Resources Preserving Center of Nanjing Agricultural University, China. The field trials were conducted at two experimental bases, Hushu (119.12° N, 31.80° E; termed E1) and Suoshi (118.89° N, 31.33° E; termed E2), in Nanjing, China. Rooted cuttings were planted in a greenhouse with an inter-plant and inter-row spacing of 0.1 m in July 2020. The plants were managed following conventional field practices.

### Trait measurements

At the end of the vegetative growth stage (late October), five plants on the inner side of the plot were selected from each accession to investigate PH-related traits, including PH (cm), IN (count), IL (cm), and SD (mm). PH was measured as the height from the base of the stem to the tip of the primary inflorescence. IN was calculated as the total number of internodes from the bottom of the stem to the primary inflorescence. IL was measured as the average internode length in the middle and upper parts. SD was measured as the diameter of the middle and upper internode. The mean value of five plants represented the trait for a certain accession.

### Statistical analysis

The broad-sense heritability (*H*^2^) was estimated using the lme4 package [10] in R software version 4.2.1. The following variance component model was used: *Y_ij_* = *μ* + *G_i_* + *Y_j_* + *GY_ij_* + *e_ij_*, where *Y_ij_* is the phenotypic value measured, *μ* is the overall mean, *G_i_* is the effect of the *i*th genotype, *Y_j_* is the effect of the *j*th environment (location), *GY_ij_* is the effect of genotype × environment interaction, and *e_ij_* is the residual effect resulting from random error. *H*^2^ was estimated according to the following equation, *H*^2^ = σ2 g/(σ2 g + σ2 ge/*n* + σ2 e/*rn*), where σ2 g is genetic variance, σ2 ge is genotype × environment interaction variance, σ2 e is residual variance, *n* is the number of environments, and *r* is the number of replications within an environment. The phenotypic values for each accession in two environments were used for further association analysis.

### Population structure and kinship analysis

Genotyping data for a panel of 346 chrysanthemum accessions (52 spray cut types, 160 disbud cut types, 60 potted and ground-cover types, 59 traditional types, and 15 wild relatives) were generated by genotyping-by-sequencing (GBS) technology, with a sequencing depth of 11× and covering 6% of the genome. The effective sequencing data were aligned to the chrysanthemum reference genome [9] using BWA [11] with the parameters mem -t 4 -k 32 –M. We then used SAMtools [12] to identify SNPs. A subset of 200 cut chrysanthemum accessions was involved in the current study for PH (Supplementary Data Table S1). The high-quality SNP marker set was filtered with a missing rate <0.2 and a minor allele frequency (MAF) >0.05. The resulting 330 710 SNPs were used for principal component analysis (PCA) and kinship analysis. PCA was performed using GCTA software [13] with default settings. The kinship matrix was calculated using the emmax-kin program in EMMAX [14].

### Genome-wide association analysis

We used 330 710 SNPs high-quality for GWAS, using a mixed linear model in EMMAX [14]. The top 10 principal components and the kinship matrix (K) were used as covariables to reduce the influence of population structure and family kinship on association analysis. Manhattan plots and quantile–quantile plots were drawn to visualize the association analysis results using the R package CMplot [15]. To avoid the loss of important loci under strict Bonferroni correction (1/effective number of independent SNPs = 4.16E−06), a less stringent empirical threshold of *P* < 1 × 10^−5^ was set to detect significant association signals [16, 17].

### Prediction of candidate genes for PH-related traits

The candidate genes were screened within a 200-kb genomic region (100 kb upstream and downstream of the significant SNPs). Candidate genes were extracted from the cultivated chrysanthemum ‘Zhongshanzigui’ reference genome [9] using BEDTools [18], and the *Arabidopsis thaliana* genomic database (www.arabidopsis.org) and the public available literature were employed to identify potential candidate genes for PH-related traits. To reveal the expression pattern of the candidate genes, the transcriptome profiles of 10 tissues, including leaf, root, shoot apex, stem, bud, disc floret petals (D_Pe), disc floret pistils (D_Pi), disc floret stamens (D_St), ray floret petals (R_Pe), and ray floret pistils (R_Pi), of a popular commercial disbud cut chrysanthemum cultivar, ‘Jinba’ were downloaded from our previously published study [9, 19]. The high-quality clean reads were mapped to the cultivated chrysanthemum ‘Zhongshanzigui’ reference genome by TopHat [20]. The expression levels of genes were evaluated using the number of fragments per kilobase of exon model per million mapped reads (FPKM) by HTSeq software [21]. The FPKMs of the candidate genes were converted to log_2_ data and visualized using TBtools software (https://github.com/CJ-Chen/TBtools). Then, the putative candidate genes identified for PH were preliminarily verified via quantitative real-time PCR (qRT–PCR). Apical bud samples from three tall and three short accessions were collected in the vigorous growing stage (45th day after planting). Specific primers for the qRT–PCR were designed by Primer Premier software and are listed in Supplementary Data Table S2. The qRT–PCR was performed using TB Green Premix Ex Taq II (TaKaRa, Japan) with three biological and three technical replicates for each sample. The expression levels of candidate genes were calculated with the 2^−ΔΔCT^ method [22], using *EF1α* as a reference gene. The association network for PH was constructed with Cytoscape v3.10.0 (https://cytoscape.org/).

### Selective sweep analysis

To determine the regions under selection for PH, the 20 tallest and shortest accessions were selected as the tall and short groups, respectively, to perform the selective sweep analysis. We calculated the nucleotide diversity (π) ratios and genetic differentiation (*F*_ST_) between the tall and short groups with a sliding window approach (100-kb sliding window and a step size of 10 kb) using the VCFtools package [23]. Windows in the top 1% of *F*_ST_ and π ratios values were considered putative selective regions.

Furthermore, selective sweep analysis was also performed using the cross-population composite likelihood ratio (XP-CLR) [24] with the same sliding window as *F*_ST_ and π ratios, and the regions with a score in the top 1% were chosen as selective sweeps. In order to exclude false positives, the overlapping candidate selection regions with three methods were used to explore potential selected genes.

### Gene Ontology functional annotations

Function annotation of candidate genes were performed with Gene Ontology (GO) (http://geneontology.org). Moreover, enrichment analysis of annotated genes was performed using OmicShare tools (https://www.omicshare.com/tools), a free online platform for data analysis.

### Genomic selection analysis

A ridge regression best linear unbiased predictor model (rrBLUP) [25] was employed for GS analysis using the package rrBLUP [26] in R software version 4.2.1. The rrBLUP model is y = WGu + ε, where y is a phenotype vector, u ~ *N* (0, Iσ^2^_u_) indicates a vector of marker effects, W represents the design matrix linking the genotype to the phenotype, G shows the genetic matrix, and ε represent random errors. The solution of the model is û = Z′(ZZ′ + λI)^−1^y, where *Z* = WG and the ridge parameter λ = σ^2^_e_/σ^2^_u_ refers to the ratio of the residual variance to marker effect variance.

The genomic prediction was evaluated with different marker sets to obtain the appropriate SNPs with high prediction accuracy for four PH-related traits. The first marker set was 330 710 SNPs used for GWAS. We ranked SNP markers from low to high according to the *P* value calculated by GWAS. The other two sets, associated500 and associated1000, included the top 500 and 1000 associations, respectively. Similarly, the two sets random500 and random1000 comprised randomly selected 500 and 1000 SNPs from 330 710 SNPs. The sigGWAS set contained SNPs reaching the GWAS significance threshold (*P* < 1 × 10^−5^). Genomic selection was performed using 5-fold cross-validation with 500 repeats. All individuals were randomly divided into five groups, of which four were regarded as the training set and one as the testing set. The prediction accuracy was evaluated by calculating Pearson’s correlation coefficient between observations and GEBVs of the testing set.

## Supplementary Material

Web_Material_uhad236

## Data Availability

The GBS data used in this study have been deposited in the National Center of Biotechnology Information Sequence Read Archive (SRA) under BioProject accession number PRJNA1004079. The data supporting this work are available in the paper and its supplementary information files. The data generated and the analytical results of the study are available from the corresponding author upon request.
