# Supplementary figures and images for: Genetic architecture and genomic prediction of plant height-related traits in chrysanthemum
Source: Hortic Res. 2023 Nov 14;11(1):uhad236. doi: 10.1093/hr/uhad236 (PMC10782495; doi:10.1093/hr/uhad236)

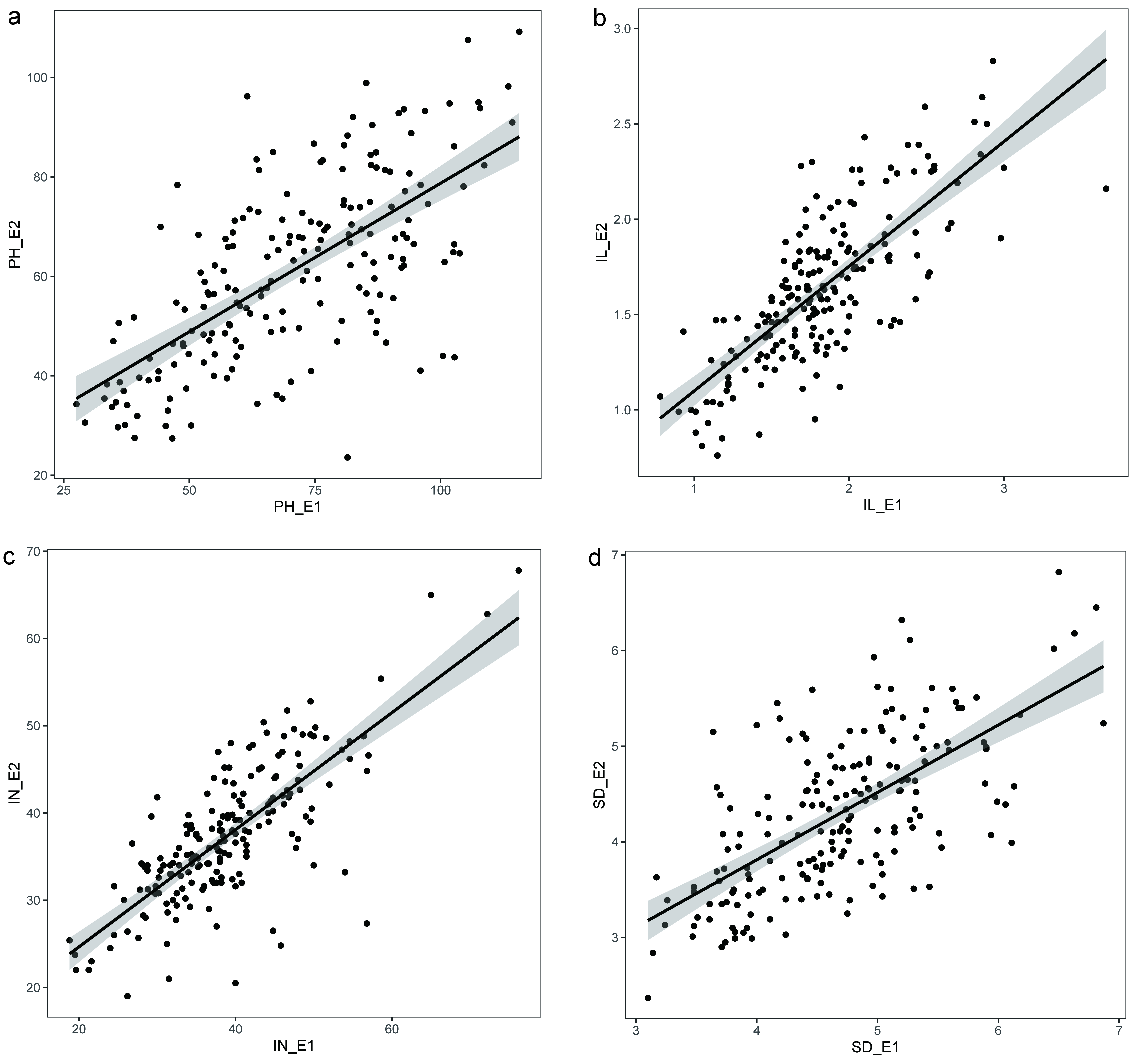

Supplement: Web_Material_uhad236 [file web_material_uhad236.zip › Fig. S1.tif]

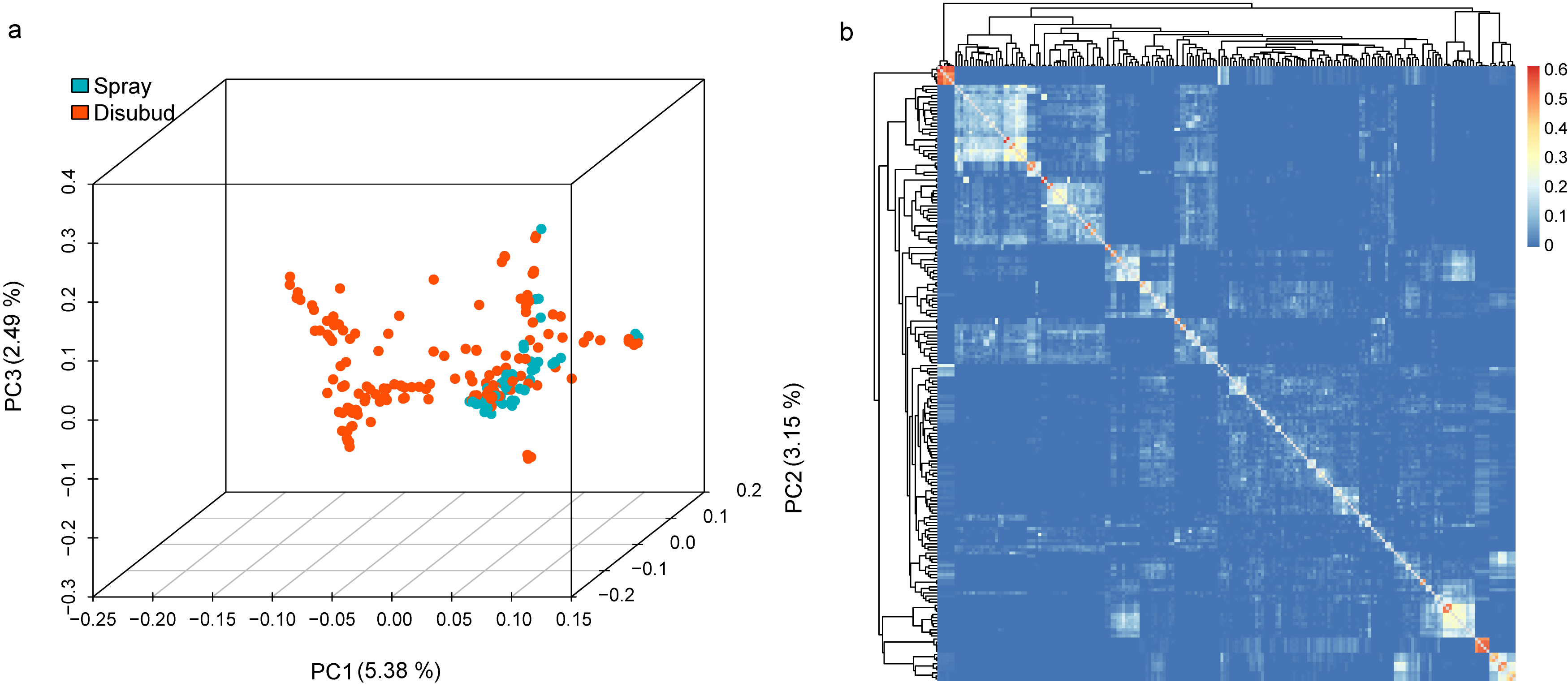

Supplement: Web_Material_uhad236 [file web_material_uhad236.zip › Fig. S2.tif]

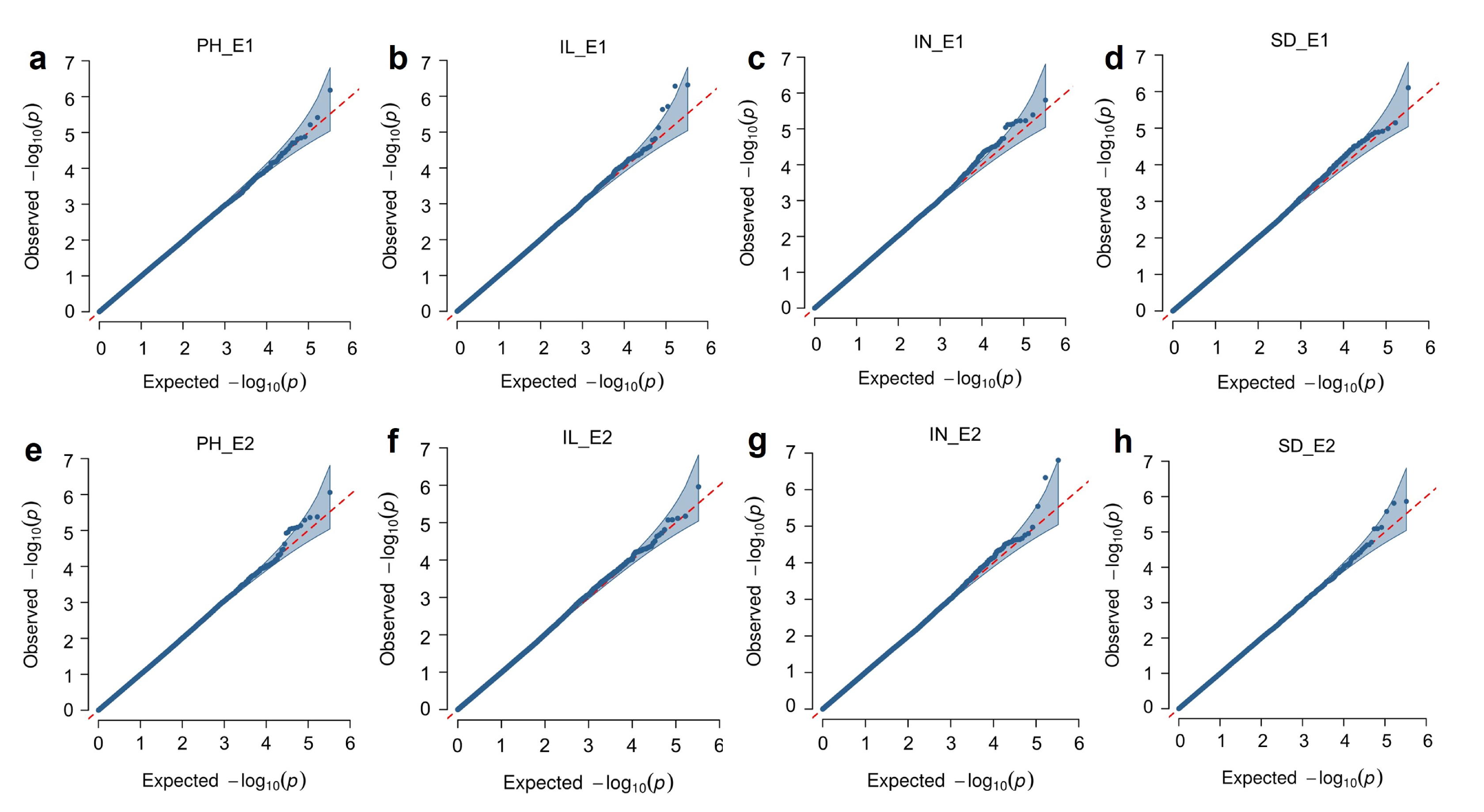

Supplement: Web_Material_uhad236 [file web_material_uhad236.zip › Fig. S3.tif]

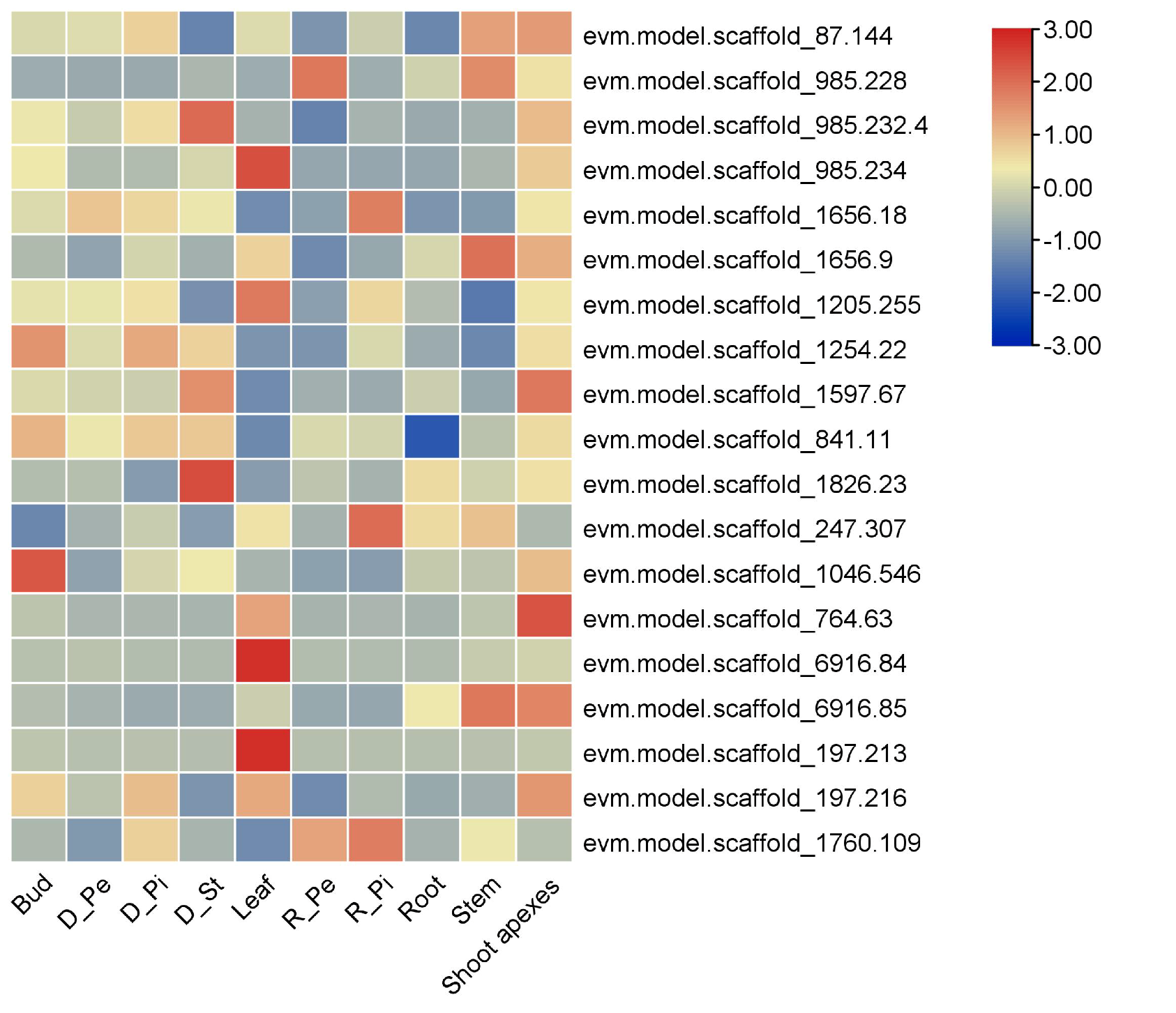

Supplement: Web_Material_uhad236 [file web_material_uhad236.zip › Fig. S4.tif]

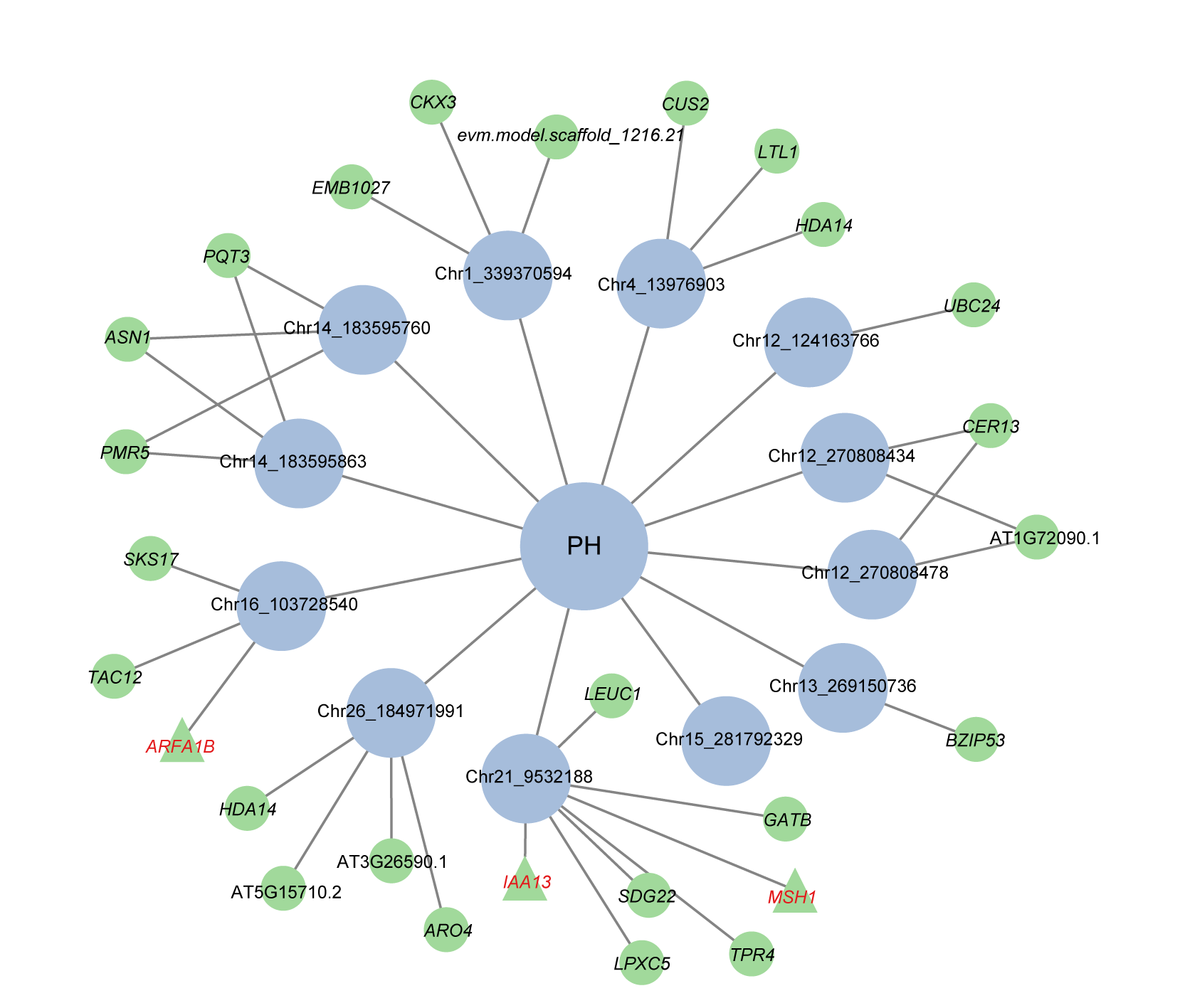

Supplement: Web_Material_uhad236 [file web_material_uhad236.zip › Fig. S5.tif]

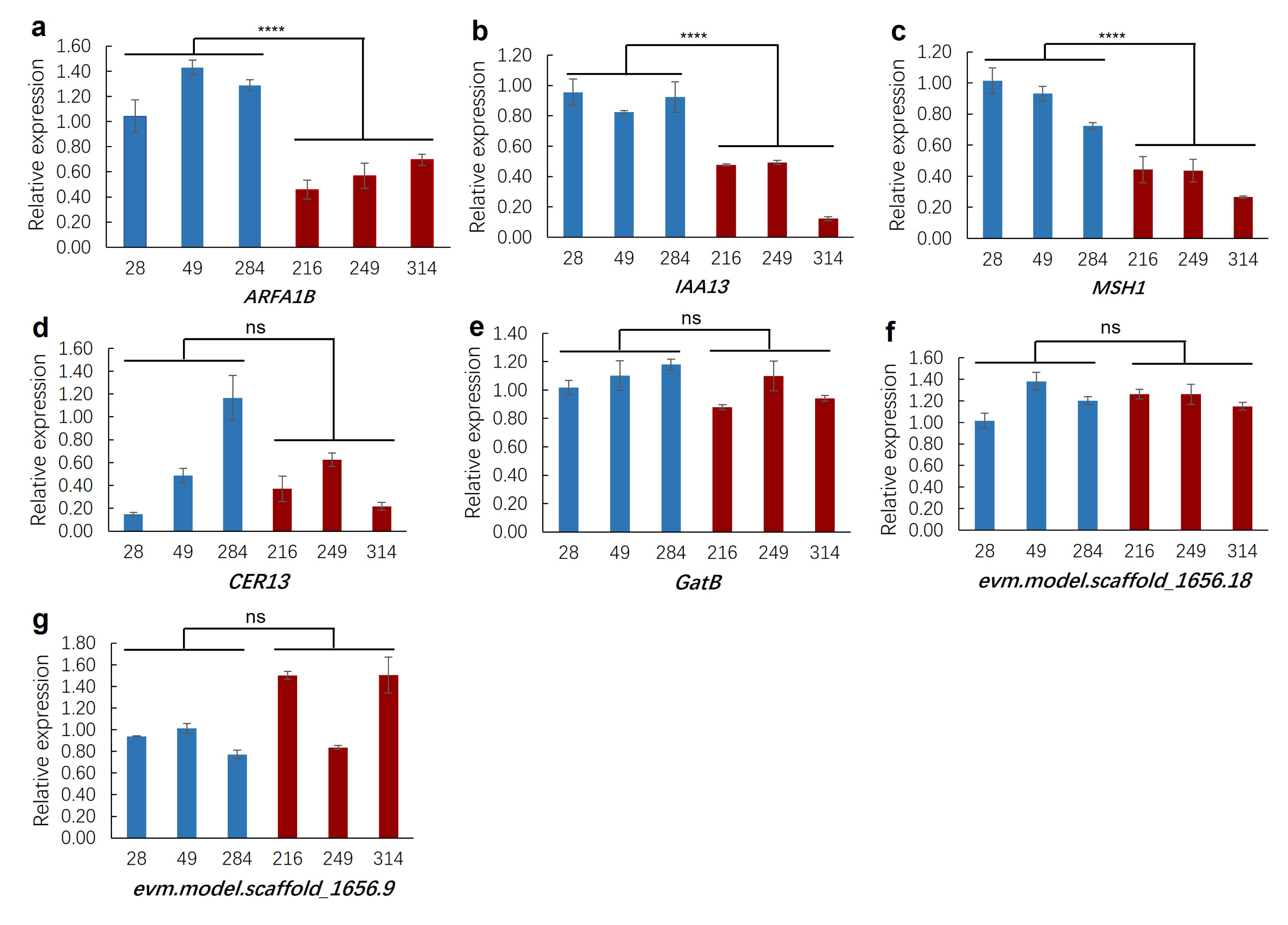

Supplement: Web_Material_uhad236 [file web_material_uhad236.zip › Fig. S6.tif]

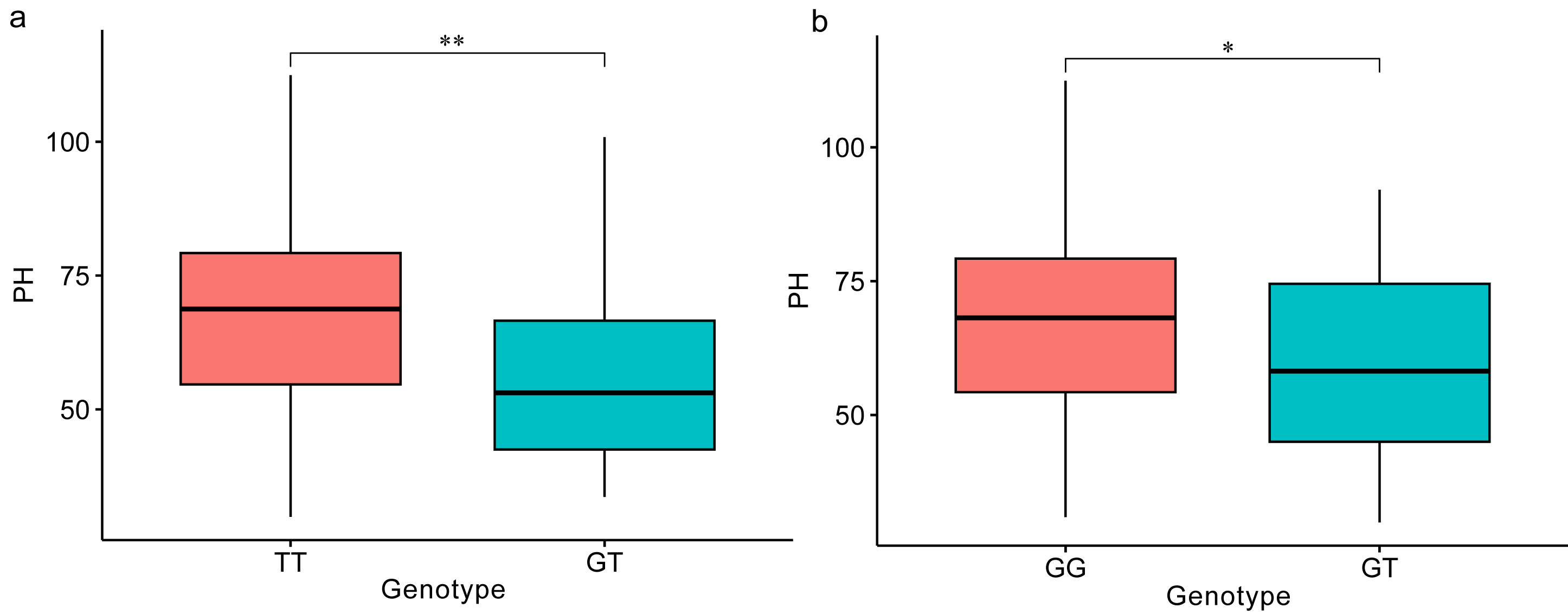

Supplement: Web_Material_uhad236 [file web_material_uhad236.zip › Fig. S7.tif]

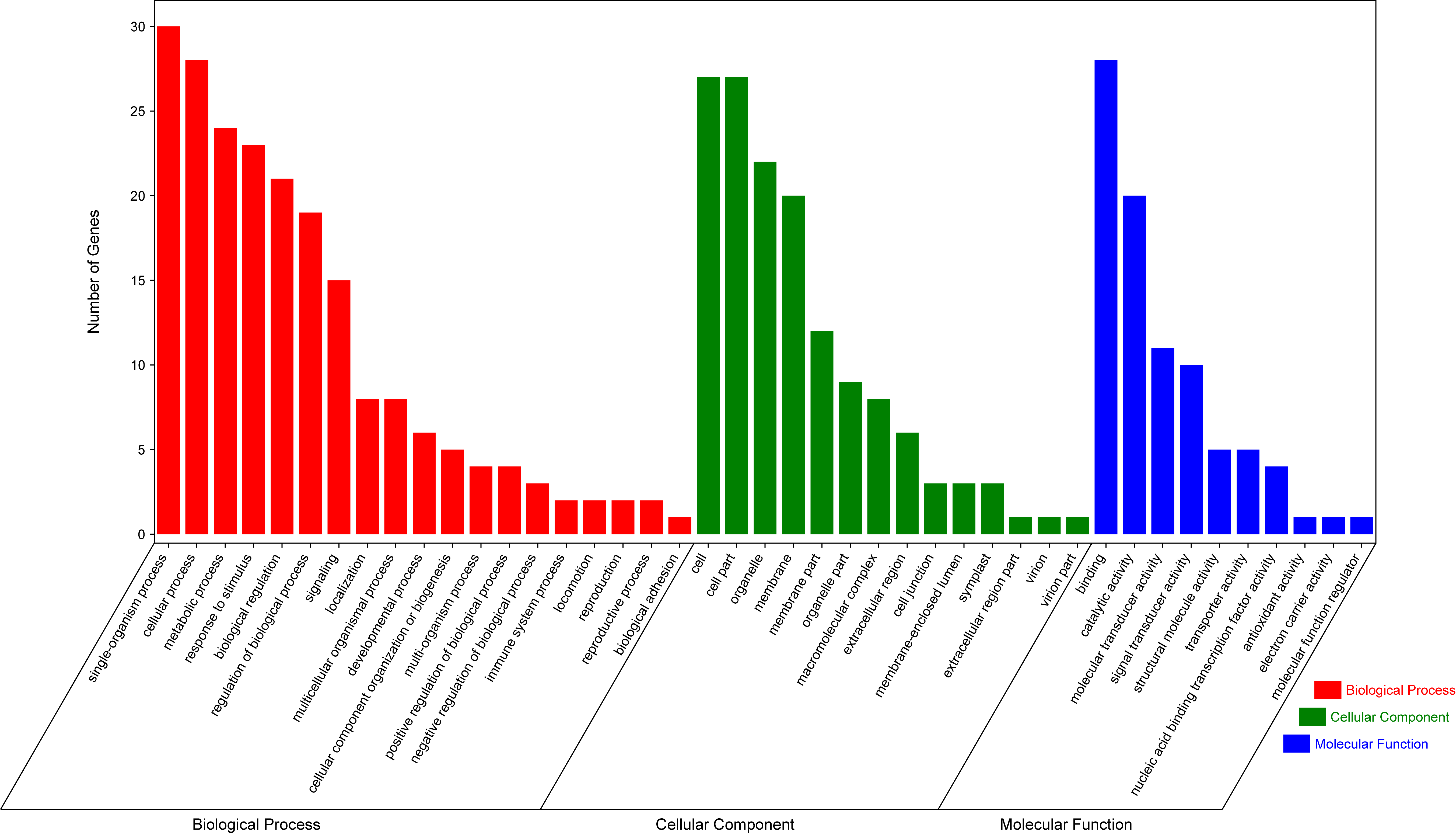

Supplement: Web_Material_uhad236 [file web_material_uhad236.zip › Fig. S8.tif]
